# Left Bundle Branch Area Pacing and Atrioventricular Node Ablation in a Single-Procedure Approach for Elderly Patients with Symptomatic Atrial Fibrillation

**DOI:** 10.3390/jcm12124028

**Published:** 2023-06-13

**Authors:** Jesse H. J. Rijks, Theo Lankveld, Randolph Manusama, Bernard Broers, Antonius M. W. van Stipdonk, Sevasti Maria Chaldoupi, Rachel M. A. ter Bekke, Ulrich Schotten, Dominik Linz, Justin G. L. M. Luermans, Kevin Vernooy

**Affiliations:** 1 Department of Cardiology, Cardiovascular Research Institute Maastricht (CARIM), Maastricht University Medical Centre (MUMC+), 6229 HX Maastricht, The Netherlands; 2 Department of Cardiology, Zuyderland Medical Centre, 6419 PC Heerlen, The Netherlands; 3 Department of Physiology, Cardiovascular Research Institute Maastricht (CARIM), Maastricht University Medical Centre (MUMC+), 6229 HX Maastricht, The Netherlands; 4 Centre for Heart Rhythm Disorders, Royal Adelaide Hospital, The University of Adelaide, Adelaide, SA 5005, Australia; 5 Department of Biomedical Sciences, Faculty of Health and Medical Sciences, University of Copenhagen, 1172 Copenhagen, Denmark

**Keywords:** atrial fibrillation, atrioventricular node ablation, left bundle branch area pacing, conduction system pacing, rate control, symptom control, elderly

## Abstract

Background: Implantation of a permanent pacemaker and atrioventricular (AV) node ablation (pace-and-ablate) is an established approach for rate and symptom control in elderly patients with symptomatic atrial fibrillation (AF). Left bundle branch area pacing (LBBAP) is a physiological pacing strategy that might overcome right ventricular pacing-induced dyssynchrony. In this study, the feasibility and safety of performing LBBAP and AV node ablation in a single procedure in the elderly was investigated. Methods: Consecutive patients with symptomatic AF referred for pace-and-ablate underwent the treatment in a single procedure. Data on procedure-related complications and lead stability were collected at regular follow-up at one day, ten days and six weeks after the procedure and continued every six months thereafter. Results: 25 patients (mean age 79.2 ± 4.2 years) were included and underwent successful LBBAP. In 22 (88%) patients, AV node ablation and LBBAP were performed in the same procedure. AV node ablation was postponed in two patients due to lead-stability concerns and in one patient on their own request. No complications related to the single-procedure approach were observed with no lead-stability issues at follow-up. Conclusions: LBBAP combined with AV node ablation in a single procedure is feasible and safe in elderly patients with symptomatic AF.

## 1. Introduction

Atrioventricular (AV) node ablation and permanent pacing is a relatively simple method for rate and symptom control in patients with atrial fibrillation (AF) and is mostly referred to as the pace-and-ablate strategy [1]. In the European Society of Cardiology 2020 guidelines for the management of atrial fibrillation, the pace-and-ablate strategy received a class IIa recommendation (should be considered) in patients unresponsive or intolerant to intensive pharmacological rate or rhythm control and not eligible for rhythm control by left atrial catheter ablation, accepting that these patients become pacemaker-dependent [1]. The treatment has been proven to be safe with a low complication rate and a better symptom reduction and quality of life compared to pharmacological rate control only [2]. One of the downsides of the therapy, when applying conventional right ventricular pacing (RVP), is the resulting dyssynchronous activation of the heart, which can lead to a pacing-induced cardiomyopathy and possibly to heart failure [3,4,5]. Therefore, conduction system pacing, including left bundle branch area pacing (LBBAP), is currently establishing as a preferred pacing modality in patients with an expected high pacing percentage [6,7,8,9]. Anticipating the potential risk of lead dislodgement during the first weeks after pacemaker implantation in patients becoming pacemaker-dependent after the AV node ablation, the pace-and-ablate strategy routinely consists of two separate procedures, the implantation of a permanent pacemaker (PM) and subsequently the catheter ablation of the AV node 4–6 weeks later [1]. Due to the low reported complication rates in LBBAP regarding lead dislocation [6,10], it might be feasible and safe to perform both PM implantation and AV node ablation procedures combined in a single procedure. 

Therefore, the aim of the present study was to investigate the feasibility and safety of performing the pace-and-ablate strategy with left bundle branch area pacing for symptomatic atrial fibrillation in elderly patients in a single-procedure approach.

## 2. Methods

This study was performed at the Maastricht University Medical Centre (MUMC+, Maastricht, The Netherlands) as a prospective study in patients undergoing a pace-and-ablate strategy for symptomatic atrial fibrillation. The local ethics committee and institutional review board approved the study (METC 2019-1313). The study complied with the Declaration of Helsinki.

### 2.1. Patient Selection

Consecutive patients referred for pace-and-ablate strategy as therapy for symptomatic AF refractory to pharmacological rate- and rhythm-control therapy or deemed unsuitable for rhythm control by left atrial catheter ablation between July 2021 and November 2022 were included. All patients received an echocardiogram <6 months before PM implantation. Patients with a class I indication for cardiac resynchronization therapy (CRT) due to systolic LV dysfunction were excluded [11]. All patients provided written informed consent.

### 2.2. Procedure

#### 2.2.1. Pacemaker Implantation

**Figure 1:**
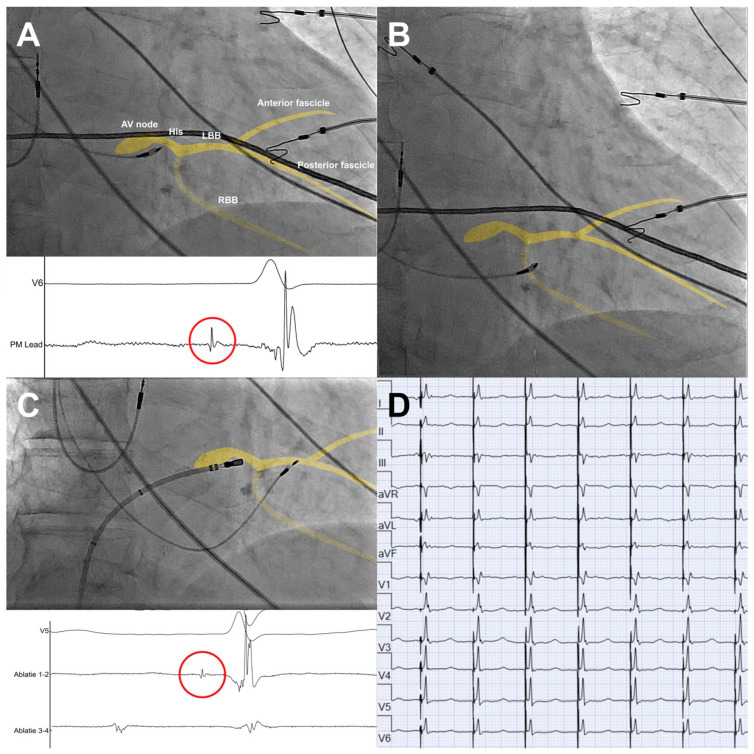
Pacemaker implantation and AV node ablation with schematic overlay of the conduction system in yellow. (**A**) Location of the AV node/bundle of His on RAO 20° view and local EGM (paper speed 200 mm/s), with His potential (red circle) recorded from the tip of the pacemaker lead. (**B**) Pacemaker lead at region of interest at the right side of the interventricular septum (IVS) ~1–1.5 cm distal from AV node/bundle of His, prior to advancing the lead through the IVS. (**C**) AV node ablation after positioning of the pacemaker lead at the left bundle branch area, including local EGM with His potential (red circle) recorded from the tip of the ablation catheter. (**D**) Final paced ECG after pacemaker implantation and AV node ablation. AV = atrioventricular, LBB = left bundle branch, RBB = right bundle branch.

All included patients underwent a pacemaker implantation with LBBAP using the SelectSecure lead (model 3830, Medtronic, Minneapolis, MN, USA). LBBAP was performed as described previously [12,13]. In short, the lead was positioned using the C315 His sheath (Medtronic, Minneapolis, MN, USA) in the region of the bundle of His in right anterior oblique 20–25° view. An intracardiac electrogram was recorded from the tip of the lead to locate the bundle of His as a reference point (Figure 1A). After identifying the bundle of His, the sheath and lead were advanced ~1–1.5 cm towards the right ventricular (RV) apex (Figure 1B). Pacemapping at the right side of the interventricular septum (IVS) was used to confirm the region of interest for LBBAP, preferably resulting in a paced left bundle branch block (LBBB) morphology with a negative, notched QRS in lead V1, a positive QRS in lead II and a negative QRS in lead III. Subsequently, the lead was advanced to the left side of the IVS. A paced QRS complex with an r′ in lead V1 was indicative of LBBAP (Figure 1D). Left bundle branch (LBB) capture was defined as: (1) an output-dependent transition in QRS morphology, either from non-selective left bundle branch pacing (LBBP) to selective left bundle branch pacing or from non-selective LBBP to left ventricular septal pacing (LVSP) at decremental voltage output pacing; (2) delay of the left bundle branch potential to V6RWPT (time to peak R wave in V6) in intrinsic rhythm equal to pacing stimulus to V6RWPT during pacing; (3) short and stable paced stimulus to V6 RWPT <75 ms in narrow QRS and <80 ms in LBBB/interventricular conduction delay (IVCD); (5) V6-V1 interpeak time > 44 ms [14]. 

#### 2.2.2. AV Node Ablation

Directly following the LBBAP implantation, patients underwent AV node ablation, unless the implanting cardiologist deemed it not safe to perform the procedure in one session due to difficulties during the implantation, such as lead dislocation, difficulties positioning the LBBAP lead, an unstable/high pacing threshold or unstable/low sensing values. Cases where the AV node ablation was not performed in the same session were also included in the final analyses.

AV node ablation was performed under fluoroscopic guidance. After administration of 5000 IE heparin, a cooled-tip RF ablation catheter (30 Watt) was positioned in the region of the compact AV node in the right atrium via femoral venous access (Figure 1C). The procedure was considered successful when there was a persistent AV block after a waiting period of 15 min with stable LBBAP parameters. All patients were planned for next-day discharge after pacemaker check-up to confirm lead stability and stable pacing parameters.

### 2.3. Data Collection

The demographic data and medical history of patients were collected after enrolment. Procedure-related data, including procedure success, procedure performed in one session and acute procedure-related complications, were collected. Follow-up data were collected at regular pacemaker follow-up one day post-implantation, as well as ten days and six weeks after implantation. Thereafter, follow-up was continued every six months. Follow-up data included pacing parameters such as the pacing threshold (volts @ 0.4 ms), sensing (millivolts) and impedance (Ohms). 

### 2.4. Safety Endpoints

Data on procedure-related complications, such as haematoma, pneumo- and haematothorax, lead perforation and cardiac tamponade, were collected. Device- and lead-related complications during follow-up, such as lead dislocation or dysfunction, lead perforation and infection, were also collected at any time during follow-up. A major complication was classified as any procedure-related complication requiring (prolonged) hospitalization, reintervention or resulting in death. A minor complication was classified as any other procedure-related complication. Furthermore, non-procedure-related serious adverse events were collected when they resulted in (prolonged) hospitalization or death.

### 2.5. Statistical Analysis

Statistical analyses were performed using IBM SPSS statistics software version 26 (SPSS Inc., Chicago, IL, USA). Continuous variables are presented as a mean ± SD when the criteria for normal distribution were met and as a median [interquartile range] when the criteria for normal distribution were not met. Discrete variables are presented as count and proportion (%). Normally distributed continuous variables were compared using a paired-samples *t*-test. Non-normally distributed continuous variables were compared using a Related-Samples Wilcoxon signed rank test. A *p*-value < 0.05 was considered statistically significant.

## 3. Results

### 3.1. Patient Characteristics

Table 1 shows the baseline characteristics of the 25 patients included in this study. The patient cohort was predominantly female (60%) with a mean age of 79.2 ± 4.2 years. The majority of patients (21 patients, 84%) were diagnosed with persistent atrial fibrillation. Patients were highly symptomatic, with EHRA class IIb or higher in 19 (76%) patients. Twelve patients were using anti-arrhythmic drugs (48%). All patients were treated with oral anti-coagulation. Fifty-two percent had undergone a previous left-sided AF ablation. The baseline mean left ventricular ejection fraction (LVEF) was 53 ± 7% with a mean left-atrial volume index (LAVI) of 53 ± 19 mL/m^2^.

### 3.2. Procedure

All patients underwent successful pacemaker implantation for LBBAP, with evidence of LBB capture in 18 (72%) patients, leaving the remaining patients with LV septal pacing. In 22 (88%) patients, AV node ablation was performed directly after LBBAP implantation. AV node ablation was postponed in two patients because of lead-stability concerns due to lead dislocation during slitting requiring immediate repositioning of the LBBAP lead. AV node ablation was postponed in one patient on request because of back complaints during PM implantation not related to the procedure. No acute procedure-related complications were observed. All patients were discharged the next morning after the lead stability and normal pacemaker function were confirmed at pacemaker check-up.

#### Follow-up

All patients were followed for a median of 81 (47–235) days, as shown in Table 2. No major procedure-related complications were observed during follow-up. One patient developed a pocket hematoma that resolved without intervention or hospitalization. The pocket hematoma might be associated with the single-procedure approach, as 5000 IE of heparin were administered for the AV node ablation, although this did not result in hospitalization or reintervention and resolved spontaneously. No PM-lead-related complications were observed during follow-up, in particular no lead dislocation or pacing and sensing issues. One patient was hospitalized for three days due to fever two weeks post-procedure. There was no evidence of a device infection, and no other focus for infection was identified (Table 2).

**Figure 2:**
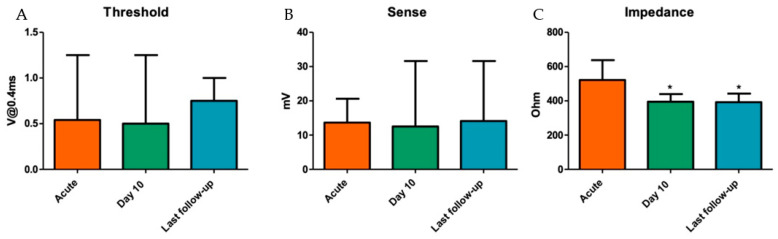
LBBAP lead pacing threshold (**A**), sense (**B**) and impedance (**C**) during FU. V = volt; mV = millivolts; * *p* < 0.01 compared to acute values.

The three patients not treated in a single procedure underwent successful AV node ablation four to six weeks after PM implantation, without any pacemaker-related complications. One patient showed re-conduction of the AV node at one day follow-up, requiring a re-ablation. No lead-stability issues occurred at follow-up. The capture-threshold and sensing-amplitude values at the last follow-up of 81 (47–235) days were stable when compared to the implant parameters: 0.5 [0.5–0.5] V @ 0.4 ms vs. 0.75 [0.5–0.75] V @ 0.4 ms (*p* = 0.075) and 14 [9,10,11,12,13,14,15,16,17,18,19] mV vs. 17 [12,13,14,15,16,17,18,19,20,21,22,23] mV (*p* = 0.31), respectively. The impedance decreased significantly during follow-up, with 522 ± 116 Ohm at implantation and 391 ± 49 Ohm at the last follow-up (*p* < 0.01, Figure 2). ECG at the last follow-up showed evidence of LBBAP (r′ in lead V1) in 24 (96%) patients. One patient revealed loss of LBBAP at follow-up. Loss of LBBAP in this patient occurred at eight months follow-up, as the ECGs at ten days and six weeks follow-up showed evidence of LBBAP. Nevertheless, the pacing threshold and sensing values remained stable, and the patient was considered for treatment with deep septal pacing. 

## 4. Discussion

This study shows the feasibility and, maybe even more importantly, the safety of performing LBBAP combined with catheter AV node ablation in a single procedure in elderly patients with symptomatic atrial fibrillation. No major complications related to the single-procedure approach were observed, and PM lead parameters remained stable during follow-up. Moreover, the patients with a delayed AV node ablation due to lead-stability-related concerns at implantation did not suffer from any pacemaker-related complications, with stable pacing and sensing values at follow-up.

### 4.1. AV Node Ablation and Permanent Pacing in Symptomatic Atrial Fibrillation

The effectiveness of PM implantation combined with AV node ablation in symptomatic atrial fibrillation has been shown before [2]. The therapy results in an improvement in symptoms and quality of life when compared to pharmacological rhythm control alone [2,15]. In the absence of systolic dysfunction, conventional RVP is recommended [1,2,11]. However, RVP results in a non-physiological dyssynchronous activation of the left ventricle (LV), which can lead to a pacing-induced cardiomyopathy in up to 20% of patients with a ventricular pacing percentage >20% [5]. This is associated with an increased incidence of heart failure (HF) and mortality [3,4]. Biventricular pacing (BiVP) has been proposed to overcome the negative effects of RVP. BiVP is achieved by the simultaneous stimulation of the RV and LV by means of an additional epicardial LV lead implanted in a coronary vein tributary. In AF patients without HF undergoing AV node ablation, BiVP was not associated with better survival compared to conventional RVP [16]. On the other hand, in patients with established heart failure, BiVP with AV node ablation was superior to pharmacological therapy alone in reducing mortality in patients with atrial fibrillation, irrespective of baseline LVEF [17]. Although BiVP theoretically ensures a more synchronous activation of the LV than RVP, it requires the placement of an extra lead in the coronary sinus, rendering the procedure more time-consuming and complex, with increased complication rates [18]. Moreover, the epi- to endocardial ventricular activation remains non-physiological [19]. Finally, the additional lead and biventricular pacing device will result in extra costs. 

Conduction system pacing (CSP) by His bundle pacing (HBP) and LBBAP was introduced as a more physiological alternative to RVP and BiVP. It has been shown that LBBAP is a feasible and safe pacing technique [6,13]. LBBAP improves left ventricular dyssynchrony considerably when compared to RVP, with levels of synchrony similar to normal intrinsic left ventricular activation [20]. Both HBP and LBBAP are associated with reduced mortality, reduced heart-failure hospitalizations and upgrades to BiVP when compared to RVP in a bradycardia population in non-randomized trials [9,21]. Vijayaraman et al. show that CSP (HBP and LBBAP) is feasible, safe and may be associated with improvement in the combined endpoint of all-cause mortality and heart-failure hospitalization when compared to RVP patients undergoing a PM implantation and AV node ablation for symptomatic atrial fibrillation in a non-randomized on-treatment comparison [22]. This study did not focus on a single-procedure approach.

### 4.2. Combining LBBAP with AV Node Ablation

Although HBP is theoretically the most physiological approach to ventricular pacing, it is associated with relatively low implantation success rates (80–85%) [23], increased pacing thresholds and sensing issues. This frequently results in a third back-up lead being placed in the RV, faster generator depletion, and both ventricular undersensing and atrial oversensing [24]. 

On the contrary, LBBAP is associated with lower pacing thresholds and higher sensing values when compared to HBP [25], and lead failure or lead dislocation is rare in LBBAP [6,13]. Moreover, the ablation site for AV node ablation is close to the pacing site of HBP, which provides an added risk for a rise in pacing thresholds. Particularly in the case of pacemaker leads placed in the proximal part of the bundle of His, AV node ablation is associated with a rise in pacing thresholds [26]. Furthermore, the use of a non-irrigated ablation catheter is recommended in HBP to avoid large lesions [27], although an irrigated ablation catheter might be needed to achieve deeper lesions in patients with a deeper anatomical location of the AV node [27], with the added risk of rising pacing thresholds. As seen in Figure 1C, the pacing site in LBBAP is located ~1–1.5 cm distal to the ablation site for AV node ablation. This causes no risk of rising pacing thresholds when performing the AV node ablation. 

Pillai et al. showed that the combination of LBBAP and AV node ablation is associated with a higher success rate and fewer acute and chronic lead-related complications than the combination of HBP and AV node ablation [28]. These beneficial aspects make LBBAP combined with AV node ablation a more attractive alternative than HBP combined with AV node ablation. LBBAP may lead to a lower threshold for applying the pace-and-ablate strategy in patients with symptomatic atrial fibrillation, since it is a pacing modality with high implantation success rates [6], low and stable pacing thresholds without sensing issues [6,29], a low risk of lead failure and lead dislocation, and, even more importantly, a near-physiological activation of the left ventricle [6,13,30]. As shown in two recent surveys on conduction system pacing, LBBAP already seems to be the preferred pacing method in pace-and-ablate strategy [7,8]. 

Previous studies on LBBAP combined with AV node ablation have been performed [22,28,31]. However, these studies did not investigate pace-and-ablate therapy with LBBAP as a single-procedure approach. Both studies by Vijayaraman et al. included patients treated with either HBP or LBBAP [22,31]. The study comparing conduction system pacing with conventional RVP reported that AV node ablation was ideally performed in one session, but this was not necessary for inclusion in the trial [22]. Pillai et al. compared HBP with LBBAP, but only 23% of patients in the HBP group and 2% of patients in the LBBAP group had PM implantation and AV node ablation performed in one session [28]. All three studies included a slightly younger population than the population in this study [22,28,31]. The guidelines emphasize performing this therapy in an older population rather than in young patients [1]. Due to the higher adverse-event rates in hospitalized elderly patients [32], we suppose that this older population will benefit particularly from a single-procedure approach. Furthermore, we anticipate that the single-procedure approach will reduce costs, since hospitalization duration is reduced. Nevertheless, the broader implementation of this single-procedure approach may require adaptations of local reimbursement models, correcting for the financial loss of income for hospitals if two billable interventions (PM implantation plus catheter AV node ablation) are combined into a single procedure. 

## 5. Limitations

This study should be interpreted considering some limitations. It is a prospective, observational study with a limited number of patients. However, our findings are in line with earlier, larger studies on LBBAP lead stability and LBBAP lead-related complications [6,29].

## 6. Conclusions

Performing left bundle branch area pacing combined with AV node ablation in a single procedure in elderly patients with symptomatic atrial fibrillation is feasible and safe. This approach reduces the burden for elderly patients to one hospital stay, which may reduce both complications associated with the hospitalization of the elderly and costs.

## Figures and Tables

**Table 1:** Baseline characteristics. BMI = body mass index, DMII = diabetes mellitus type II, COPD = chronic obstructive lung disease, CVA/TIA = cerebrovascular accident/transient ischaemic attack, ACEi/ARB = angiotensin-converting enzyme inhibitor/angiotensin receptor blocker, MRA = mineralocorticoid receptor antagonist, CCB = calcium channel blocker, AAD = anti-arrhythmic drug, OAC = oral anticoagulation, NOAC = non-vitamin K oral anticoagulation, VKA = vitamin K antagonist, LBBB = left bundle branch block, LVEDD = left ventricular end-diastolic diameter, LVESD = left ventricular end-systolic diameter, LAVI = left atrial volume indexed for body surface area, AF = atrial fibrillation.

Baseline Characteristics (N = 25)	
Age, yrs ± SD	79.2 ± 4.2
Female, *n* (%)	15 (60)
BMI, kg/m^2^ ± SD	27.0 ± 4.6
**Comorbidities**	
DMII, *n* (%)	2 (8)
Hypertension, *n* (%)	17 (68)
COPD, *n* (%)	3 (12)
CVA/TIA, *n* (%)	4 (16)
Coronary artery disease, *n* (%)	3 (12)
**Atrial fibrillation**	
Paroxysmal, *n* (%)	1 (4)
Persistent, *n* (%)	21 (84)
Longstanding persistent, *n* (%)	3 (12)
**Symptoms**	
EHRA I, *n* (%)	0 (0)
EHRA IIa, *n* (%)	6 (24)
EHRA IIb, *n* (%)	14 (56)
EHRA III, *n* (%)	5 (20)
EHRA IV, *n* (%)	0 (0)
**Medication**	
Beta-blocker, *n* (%)	13 (52)
ACEi/ARB, *n* (%)	14 (56)
MRA, *n* (%)	3 (12)
Digoxin, *n* (%)	5 (20)
Dihydropyridine CCB, *n* (%)	5 (20)
Non-dihydropyridin CCB, *n* (%)	6 (24)
Class I/III AAD, *n* (%)	12 (48)
Flecainide, *n* (%)	2 (8)
Sotalol, *n* (%)	5 (20)
Amiodarone, *n* (%)	5 (20)
OAC	25 (100)
DOAC, *n* (%)	23 (92)
VKA, *n* (%)	2 (8)
QRS duration, ms [IQR]	92 (87–98)
LBBB, *n* (%)	2 (8)
Ejection fraction, % ± SD	53 ± 7
LVEDD mm ± SD	47 ± 6
LVESD mm ± SD	34 ± 5
LAVI, ml/m^2^ ± SD	53 ± 19
Previous AF ablation, *n* (%)	13 (52)

**Table 2:** Follow-up.

Complications	
Follow-up duration, days [IQR]	81 (47–235)
Complication, n (%)	1 (4)
Major ^^^	0
Minor	1 *
Serious adverse event, n (%)	1 (4) ^#^

^ complication requiring hospitalization or reintervention, resulting in death. * pocket hematoma, resolved without intervention or hospitalization. ^#^ fever requiring hospitalization, without evidence of pacemaker infection.

## Data Availability

The data presented in this study are available on request from the corresponding author. The data are not publicly available due to ethical and privacy restrictions.

## References

[1] Hindricks G., Potpara T., Dagres N., Arbelo E., Bax J.J., Blomström-Lundqvist C., Boriani G., Castella M., Dan G.-A., E Dilaveris P. (2021). 2020 ESC Guidelines for the diagnosis and management of atrial fibrillation developed in collaboration with the European Association for Cardio-Thoracic Surgery (EACTS): The Task Force for the diagnosis and management of atrial fibrillation of the European Society of Cardiology (ESC) Developed with the special contribution of the European Heart Rhythm Association (EHRA) of the ESC. Eur. Heart J..

[2] Lim K.-T., Davis M.J., Powell A., Arnolda L., Moulden K., Bulsara M., Weerasooriya R. (2007). Ablate and pace strategy for atrial fibrillation: Long-term outcome of AIRCRAFT trial. Europace.

[3] Wilkoff B.L., Cook J.R., Epstein A.E., Greene H.L., Hallstrom A.P., Hsia H., Kutalek S.P., Sharma A. (2002). Dual-chamber pacing or ventricular backup pacing in patients with an implantable defibrillator: The Dual Chamber and VVI Implantable Defibrillator (DAVID) Trial. JAMA.

[4] Sweeney M.O., Hellkamp A.S., Ellenbogen K.A., Greenspon A.J., Freedman R.A., Lee K.L., Lamas G.A., Investigators MOST (2003). Adverse effect of ventricular pacing on heart failure and atrial fibrillation among patients with normal baseline QRS duration in a clinical trial of pacemaker therapy for sinus node dysfunction. Circulation.

[5] Khurshid S., Epstein A.E., Verdino R.J., Lin D., Goldberg L.R., Marchlinski F.E., Frankel D.S. (2014). Incidence and predictors of right ventricular pacing-induced cardiomyopathy. Heart Rhythm..

[6] Jastrzębski M., Kiełbasa G., Cano O., Curila K., Heckman L., De Pooter J., Chovanec M., Rademakers L., Huybrechts W., Grieco D. (2022). Left bundle branch area pacing outcomes: The multicentre European MELOS study. Eur. Heart J..

[7] Keene D., Anselme F., Burri H., Pérez C., Čurila K., Derndorfer M., Foley P., Gellér L., Glikson M., Huybrechts W. (2023). Conduction system pacing, a European survey: Insights from clinical practice. Europace.

[8] Kircanski B., Boveda S., Prinzen F., Sorgente A., Anic A., Conte G., Burri H. (2023). Conduction system pacing in everyday clinical practice: EHRA physician survey. Europace.

[9] Sharma P.S., Patel N.R., Ravi V., Zalavadia D.V., Dommaraju S., Garg V., Larsen T.R., Naperkowski A.M., Wasserlauf J., Krishnan K. (2022). Clinical outcomes of left bundle branch area pacing compared to right ventricular pacing: Results from the Geisinger-Rush Conduction System Pacing Registry. Heart Rhythm..

[10] Li X., Li H., Ma W., Ning X., Liang E., Pang K., Yao Y., Hua W., Zhang S., Fan X. (2019). Permanent left bundle branch area pacing for atrioventricular block: Feasibility, safety, and acute effect. Heart Rhythm..

[11] Glikson M., Nielsen J.C., Kronborg M.B., Michowitz Y., Auricchio A., Barbash I.M., Barrabés J.A., Boriani G., Braunschweig F., Brignole M. (2021). 2021 ESC Guidelines on cardiac pacing and cardiac resynchronization therapy. Eur. Heart J..

[12] Huang W., Chen X., Su L., Wu S., Xia X., Vijayaraman P. (2019). A beginner’s guide to permanent left bundle branch pacing. Heart Rhythm..

[13] Heckman L.I.B., Luermans J.G.L.M., Jastrzębski M., Weijs B., Van Stipdonk A.M.W., Westra S., Uijl D.D., Linz D., Mafi-Rad M., Prinzen F.W. (2022). A single-centre prospective evaluation of left bundle branch area pacemaker implantation characteristics. Neth. Heart J..

[14] Burri H., Jastrzebski M., Cano Ó., Čurila K., de Pooter J., Huang W., Israel C., Joza J., Romero J., Vernooy K. (2023). EHRA clinical consensus statement on conduction system pacing implantation: Endorsed by the Asia Pacific Heart Rhythm Society (APHRS), Canadian Heart Rhythm Society (CHRS), and Latin American Heart Rhythm Society (LAHRS). Europace.

[15] Queiroga A., Marshall H.J., Clune M., Gammage M.D. (2003). Ablate and pace revisited: Long term survival and predictors of permanent atrial fibrillation. Heart.

[16] Chatterjee N.A., Upadhyay G.A., Ellenbogen K.A., Hayes D.L., Singh J.P. (2012). Atrioventricular nodal ablation in atrial fibrillation: A meta-analysis of biventricular vs. right ventricular pacing mode. Eur. J. Heart Fail..

[17] Brignole M., Pentimalli F., Palmisano P., Landolina M., Quartieri F., Occhetta E., Calò L., Mascia G., Mont L., Vernooy K. (2021). AV junction ablation and cardiac resynchronization for patients with permanent atrial fibrillation and narrow QRS: The APAF-CRT mortality trial. Eur. Heart J..

[18] Kirkfeldt R.E., Johansen J.B., Nohr E.A., Jørgensen O.D., Nielsen J.C. (2014). Complications after cardiac implantable electronic device implantations: An analysis of a complete, nationwide cohort in Denmark. Eur. Heart J..

[19] van Rees J.B., de Bie M.K., Thijssen J., Borleffs C.J.W., Schalij M.J., van Erven L. (2011). Implantation-related complications of implantable cardioverter-defibrillators and cardiac resynchronization therapy devices: A systematic review of randomized clinical trials. J. Am. Coll. Cardiol..

[20] Heckman L.I., Luermans J.G., Curila K., Van Stipdonk A.M., Westra S., Smisek R., Prinzen F.W., Vernooy K. (2021). Comparing Ventricular Synchrony in Left Bundle Branch and Left Ventricular Septal Pacing in Pacemaker Patients. J. Clin. Med..

[21] Abdelrahman M., Subzposh F.A., Beer D., Durr B., Naperkowski A., Sun H., Oren J.W., Dandamudi G., Vijayaraman P. (2018). Clinical Outcomes of His Bundle Pacing Compared to Right Ventricular Pacing. J. Am. Coll. Cardiol..

[22] Vijayaraman P., Mathew A.J., Naperkowski A., Young W., Pokharel P., Batul S.A., Storm R., Oren J.W., Subzposh F.A. (2022). Conduction system pacing versus conventional pacing in patients undergoing atrioventricular node ablation: Nonrandomized, on-treatment comparison. Heart Rhythm O2..

[23] Keene D., Arnold A.D., Jastrzębski M., Burri H., Zweibel S., Crespo E., Chandrasekaran B., Bassi S., Joghetaei N., Swift M. (2019). His bundle pacing, learning curve, procedure characteristics, safety, and feasibility: Insights from a large international observational study. J. Cardiovasc. Electrophysiol..

[24] Zanon F., Ellenbogen K.A., Dandamudi G., Sharma P.S., Huang W., Lustgarten D.L., Tung R., Tada H., Koneru J.N., Bergemann T. (2018). Permanent His-bundle pacing: A systematic literature review and meta-analysis. Europace.

[25] Hua W., Fan X., Li X., Niu H., Gu M., Ning X., Hu Y., Gold M.R., Zhang S. (2020). Comparison of Left Bundle Branch and His Bundle Pacing in Bradycardia Patients. JACC Clin. Electrophysiol..

[26] Vijayaraman P., Subzposh F.A., Naperkowski A. (2017). Atrioventricular node ablation and His bundle pacing. Europace.

[27] Muthumala A., Vijayaraman P. (2020). His-Purkinje conduction system pacing and atrioventricular node ablation. Herzschrittmacherther Elektrophysiol..

[28] Pillai A., Kolominsky J., Koneru J.N., Kron J., Shepard R.K., Kalahasty G., Huang W., Verma A., Ellenbogen K.A. (2022). Atrioventricular junction ablation in patients with conduction system pacing leads: A comparison of His-bundle vs left bundle branch area pacing leads. Heart Rhythm..

[29] Su L., Wang S., Wu S., Xu L., Huang Z., Chen X., Zheng R., Jiang L., Ellenbogen K.A., Whinnett Z.I. (2021). Long-Term Safety and Feasibility of Left Bundle Branch Pacing in a Large Single-Center Study. Circ. Arrhythm. Electrophysiol..

[30] Rijks J., Luermans J., Heckman L., van Stipdonk A.M., Prinzen F., Lumens J., Vernooy K. (2022). Physiology of Left Ventricular Septal Pacing and Left Bundle Branch Pacing. Card. Electrophysiol. Clin..

[31] Vijayaraman P., Hashimova N., Mathew A.J., Subzposh F.A., Naperkowski A. (2022). Simultaneous conduction system pacing and atrioventricular node ablation via axillary vs femoral access. Heart Rhythm..

[32] Long S.J., Brown K.F., Ames D., Vincent C. (2013). What is known about adverse events in older medical hospital inpatients? A systematic review of the literature. Int. J. Qual Health Care.

